# Is Ferroptosis a Future Direction in Exploring Cryptococcal Meningitis?

**DOI:** 10.3389/fimmu.2021.598601

**Published:** 2021-03-19

**Authors:** Xianbin Xu, Danfeng Lin, Sheng Tu, Shiqi Gao, Anwen Shao, Jifang Sheng

**Affiliations:** ^1^ State Key Laboratory for Diagnosis and Treatment of Infectious Diseases, Collaborative Innovation Center for Diagnosis and Treatment of Infectious Diseases, The First Affiliated Hospital, College of Medicine, Zhejiang University, Hangzhou, China; ^2^ Department of Surgical Oncology, Second Affiliated Hospital, School of Medicine, Zhejiang University, Hangzhou, China; ^3^ Department of Neurosurgery, Second Affiliated Hospital, School of Medicine, Zhejiang University, Hangzhou, China

**Keywords:** cryptococcal meningitis, ferroptosis, iron accumulation, lipid peroxidation, immunomodulatory activity, inflammation, infection

## Abstract

Cryptococcal meningitis (CM) is the leading cause of mortality among patients infected with human immunodeficiency virus (HIV). Although treatment strategies for CM are continually being developed, the mortality rate is still high. Therefore, we need to explore more therapeutic strategies that are aimed at hindering its pathogenic mechanism. In the field of CM, several studies have observed rapid iron accumulation and lipid peroxidation within the brain, all of which are hallmarks of ferroptosis, which is a type of programmed cell death that is characterized by iron dependence and lipid peroxidation. In recent years, many studies have confirmed the involvement of ferroptosis in many diseases, including infectious diseases such as *Mycobacterium tuberculosis* infection and coronavirus disease-2019 (COVID-19). Furthermore, ferroptosis is considered as immunogenic and pro-inflammatory as the ferroptotic cells release damage-associated molecular pattern molecules (DAMPs) and alarmin, both of which regulate immunity and pro-inflammatory activity. Hence, we hypothesize that there might be a relationship between this unique cell death modality and CM. Herein, we review the evidence of ferroptosis in CM and consider the hypothesis that ferroptotic cell death may be involved in the cell death of CM.

## Introduction

Cryptococcal meningitis (CM) is the most common lethal fungal infection in patients with acquired immune deficiency syndrome (AIDS). In 2009, Park et al. first estimated the global burden of HIV-associated *cryptococcal* infection, and identified approximately 957,900 new cases of CM per year worldwide, resulting in 624,700 deaths within three months after infection (1). Additional studies have also suggested an increasing number of annual global deaths, with CM being responsible for 15% of AIDS-related deaths (2, 3). A recent systematic review reported a total of 8,769 cases of cryptococcosis in mainland China from 1985 to 2010 through the use of CBMdisk (China Biology and Medicine data disc) database (4). However, the incidence of cryptococcosis in China is likely much higher than the reported number due to missed and misdiagnosed cases. According to the United Nations AIDS Program (UNAIDS), approximately 37.9 million people are currently living with HIV, and as HIV-infected patients have hypoimmunity, they are more prone to acquiring opportunistic infections, including CM.

CM is a type of subacute meningoencephalitis that is mainly caused by *Cryptococcus neoformans*, which largely attacks patients with immunodeficiency, and leads to the development of fungal meningitis (1, 5, 6). The main transmission route of *cryptococcal* infection is inhalation of yeast or basidiospores through the respiratory tract, though human-to-human or mother-to-child transmissions have been reported (7, 8). The main virulence factors of *C. neoformans* include production of melanin and polysaccharide capsule (9). Melanin is synthesized in *C. neoformans* by being primarily converted from catecholamines, such as dopa, dopamine, and epinephrine. As these catecholamines are neurotransmitters within the central nervous system (CNS) (10–12), *C. neoformans* have characteristics of neurotropism (13). Furthermore, several studies have demonstrated that *C. neoformans* is able to escape from attacks that are caused by the immune system by protecting melanin (10, 14). With regards to the polysaccharide capsule, it is able to exacerbate the toxicity of CM infection under some conditions, which include anti-phagocytosis, complement depletion, and inhibition of leukocyte migration (11, 12). As these are characteristics of *C. neoformans*, the most common presentation of invasive *cryptococcal* infection in patients living with HIV is lethal meningitis and meningoencephalitis.

Currently, the predominant therapies for HIV-associated CM include a combination of antifungal drugs, control of intracranial pressure, and appropriately timed initiation of highly active antiretroviral therapy (HAART) (15, 16). The treatment course involves an induction period, a consolidation period, and a maintenance period (16). Although novel treatment strategies for CM are continually being identified and developed, the mortality rate remains high due to a number of reasons, including infection of CNS, drug resistance, high cost, and availability of essential drugs. Therefore, there is a need to explore more treatment strategies and potential therapeutic targets. For instance, recent studies have shown that sertraline, a selective serotonin reuptake inhibitor antidepressant, exhibits excellent *in vitro-in vivo* antifungal activity (17–19). Some clinical trials have also identified that adjuvant sertraline can partially improve clearance of the cerebrospinal fluid (CSF) fungus (20–22). Unfortunately, a randomized, placebo-controlled, double-blind phase 3 trial confirmed that sertraline did not reduce the mortality of HIV-associated CM, which may be related to insufficient duration of therapeutic sertraline concentration (21). Moreover, it is important to keep exploring therapeutic strategies that are aimed at determining the pathogenesis of CM. Some recent studies have indicated increased iron accumulation and lipid peroxidation in CM, thus revealing a connection between CM and ferroptosis (23–27).

Recently, the role of ferroptosis in cell death has become a research hotspot and is associated with many diseases (28–30), such as tumors and ischemic organ injuries. Ferroptosis is known to play a role in various brain diseases (31–34), including stroke, Parkinson’s disease (PD), and Alzheimer’s disease (AD). There are also several studies that have shown the involvement of ferroptosis in infections caused by different pathogens, such as *Pseudomonas aeruginosa* (35, 36), *Mycobacterium tuberculosis* (37–39), severe acute respiratory syndrome coronavirus 2 (SARS CoV-2) (40–42), and hepatitis B virus (HBV) (43, 44). Beyond that, ferroptosis is associated with immunogenic and pro-inflammatory activities. Ferroptotic cells are known to release DAMPs and immune-stimulating cellular components, which can be recognized by immune receptors, thus inducing cell death and inflammation. Furthermore, ferroptosis of immune cells during an infection can have an effect on immune function and create a beneficial environment for infectious agents. Taken together, ferroptosis plays a part in brain disease and infectious diseases, and likely regulates immunity and inflammation. Importantly, ferroptosis’s key hallmarks, which include iron accumulation and lipid peroxidation, are known in CM. Therefore, we hypothesize that ferroptosis is involved in pathological process of CM, and regulating ferroptosis represents a novel therapeutic target and strategy. Herein, determining what the function of ferroptosis is in CM and how ferroptosis affects the immune system in infectious diseases remains to be resolved.

## Brief History and Mechanisms of Ferroptosis

**Figure 1:**
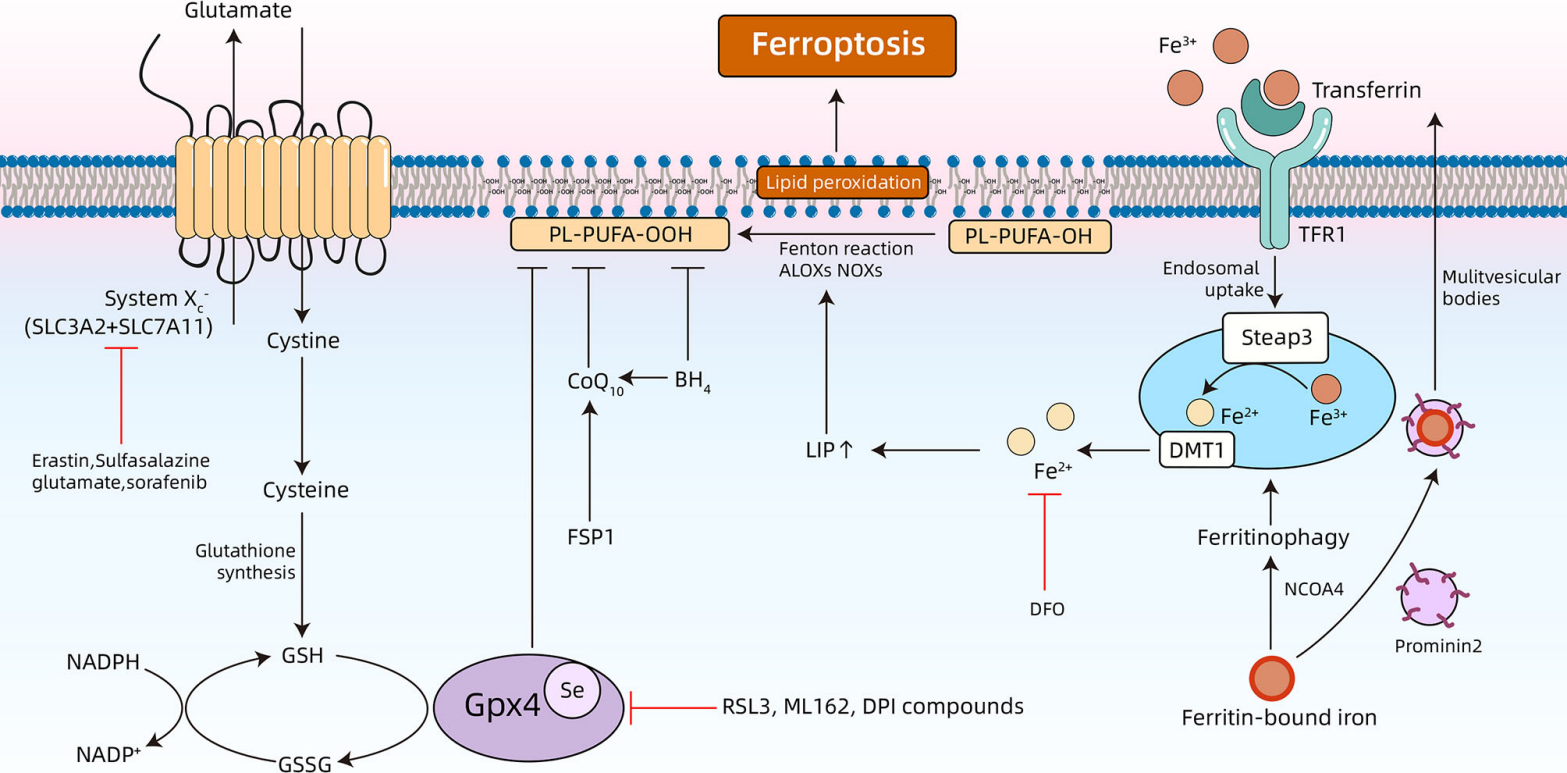
Overview of the underlying mechanisms of ferroptosis. Ferroptosis is a type of programmed cell death that is characterized by accumulation of free iron and toxic lipid peroxides. Dysregulation of intracellular iron metabolism or glutathione peroxidation pathways leads to accumulation of lipid reactive oxygen species (ROS), and eventually cell death. Various inducers and inhibitors of ferroptosis are shown. TFR1, transferrin receptors 1; DFO, deferoxamine; NCOA4, nuclear receptor coactivator 4; ALOXs: arachidonate lipoxygenases; NADPH, nicotinamide adenine dinucleotide phosphate; NADP^+^, nicotinamide adenine dinucleotide phosphate; NOXs: Reduced form of NADPH oxidase; BH4, tetrahydrobiopterin; FSP1, ferroptosis suppressor protein 1; Se, selenocysteine; GSSG, oxidized GSH.

In 2003, Dolma et al. performed a synthetic lethal high-throughput screening to investigate the ability of 23,550 compounds to kill engineered tumorigenic cells, results of which showed that a compound named erastin was able to induce a non-apoptotic cell death process (45). Subsequently, in 2008, two small molecule compounds, Ras synthetic lethal 3 (RSL3) and Ras synthetic lethal 5 (RSL5), were observed to stimulate an iron-dependent non-apoptotic cell death, and shared similar properties to erastin (46). In 2012, Dixon et al. identified that erastin-induced oxidative iron-dependent cell death was morphologically different from the characteristics of apoptosis, necrosis, and autophagy. Furthermore, this particular form of cell death was named ferroptosis (28). In the following years, three different types of metabolism, iron metabolism, lipid metabolism, and amino acid metabolism, were described as the main mechanisms of ferroptosis (**Figure 1**) (28).

### Iron Metabolism

Preliminary studies have identified that ferroptosis can be suppressed by co-treatment with iron chelators (deferoxamine), and potentiated by incubating with three different exogenous iron sources (28). These results support that iron metabolism is involved in pathogenesis of ferroptosis. Additionally, more in-depth research has validated the role of iron in ferroptosis (**Figure 1**). Extracellular Fe^3+^ bound to transferrin (Tf) are recognized by transferrin receptors (TFRs) on the cellular membrane, and imported into the cytoplasm (47). Next, Fe^3+^ is converted to Fe^2+^ in endosomes using a six-transmembrane epithelial antigen of prostate 3 (STEAP3), a ferrireductase. Then, Fe^2+^ is released from the endosomes and into the cytoplasm through divalent metal transporter 1 (DMT1), thereby increasing the concentration of the labile iron pool (LIP). Ferritinophagy refers to selective autophagy of ferritin and can modulate sensitivity to ferroptosis by adjusting LIP content (48–50). All the free iron of LIP facilitates ferroptosis by triggering formation of highly damaging hydroxyl radicals using the Fenton reaction, and catalyzing lipid peroxidation in the cellular membrane through lipoxygenase (LOXs) (51, 52). Under physiological conditions, the human body is able to maintain the homeostasis of intracellular iron. For example, intracellular iron can be effectively expelled to the outside of the cell using transferrin. Furthermore, prominin 2 can inhibit ferroptosis by promoting the formation of multivesicular bodies and exosomes (53).

### Lipid Metabolism

Ferroptosis, a process that is heavily dependent on reactive oxygen species (ROS) and iron, is characterized by lipid peroxidation (54–56). Lipid peroxidation refers to the process in which oxidants (i.e., free radicals) abstract a labile hydrogen atom from the methylene group at the bisallyl position of polyunsaturated fatty acids (PUFAs), and PUFAs are substrates of pro-ferroptotic lipid peroxidation products including ACSL4 and LPCAT3 (54). Among the different classes of lipids in cells (i.e., fatty acids, phospholipids, cholesterols, cardiolipins, and sphingolipids), PUFAs containing bis-allylic protons vulnerable to hydrogen atom abstraction are the lipids that are most susceptible to oxidative damage in ferroptosis (57). After it was first noticed that ferroptosis is induced by RSL3 and erastin, further studies confirmed that this specially programmed cell death is driven by a loss of activity of the lipid repair enzyme glutathione (GSH)-glutathione peroxidase 4 (Gpx4) (**Figure 1**). Gpx4 is the sole selenoenzyme that is able to catalyze the conversion of toxic lipid hydroperoxides (L-OOH) into non-toxic lipid alcohols (L-OH) in a complex cellular membrane environment (58, 59). The deletion and inhibition of Gpx4 is able to facilitate lethal accumulation of lipid peroxides into the cellular membrane and initiate execution of ferroptosis. Therefore, Gpx4 is considered to be a central link in regulating ferroptosis (60). Furthermore, there are novel emerging ferroptosis-inducing compounds (i.e., ML162 and DPI compounds) that are able to induce lipid peroxidation by inhibiting Gpx4 function (28, 60). Moreover, lipophilic antioxidants, which include vitamin E, ferrostatin-1 (Fer-1), and liproxstatin-1 (Lip-1), are able to alleviate ferroptosis by inhibiting lipid autoxidation (61–63).

### Amino Acid and GSH Metabolism

The core part of the amino acid-related mechanism of ferroptosis is the GSH-Gpx4 axis (**Figure 1**) (60). System $x_{c}^{-}$, a Na^+^-independent cystine/glutamate antiporter, is a disulfide-linked heterodimer that consists of SLC3A2 and SLC7A11. System $x_{c}^{-}$ imports extracellular cystine into the cytoplasm and simultaneously exports intracellular glutamate into the extracellular compartment (64, 65). The intracellular cystine will be then converted to cysteine, which is further used to synthesize GSH (66). GSH, a tripeptide antioxidant, is then used as a cofactor for Gpx4, which reduces oxidative stress and maintains the intracellular redox balance. In addition to the canonical amino acid metabolism pathway that is mentioned above, coenzyme Q10 (CoQ10) works in parallel with Gpx4 in order to suppress phospholipid peroxidation and ferroptosis. Many molecules that induce ferroptosis by regulating amino acid metabolism have now been discovered. Erastin (28), sulfasalazine (SAS) (67), and sorafenib (68) are able to inhibit system $x_{c}^{-}$, which causes GSH depletion, thereby inducing ferroptotic cell death. β-mercaptoethanol is able to promote cystine uptake in order to inhibit erastin-stimulated ferroptosis in HT1080 cells (67). Acetaminophen-induced cell death is characterized by a depletion of GSH, and ferroptosis has been shown to be involved in this process (69). Importantly, the accumulation of extracellular glutamate is a natural trigger for inducing ferroptosis (60). Previous studies have indicated that excessive stimulation of glutamate is able to induce death of nerve cells (70, 71). Glutamate-induced toxicity is an oxidative and iron-dependent process that is caused by calcium influx and competitive inhibition of system $x_{c}^{-}$, which ultimately induces ferroptosis by inhibiting Gpx4 (60, 70, 71).

## Evidence of Ferroptosis in CM

Of note, previous studies summarized below have indicated that increased iron accumulation, lipid peroxidation in CM, and abnormal activity of amino acid, which suggests a relationship between CM and ferroptosis.

### Increased Iron Accumulation in the Brain With CM

Iron is the most abundant trace metal present in organisms and is essential for both humans and pathogenic microbes (72, 73). Several central metabolic pathways, including oxygen transport, the tricarboxylic acid (TCA) cycle, and electron transport chains, cannot be done in the absence of iron (74). However, under pathological conditions, iron accumulation damages the body. Ferrous iron (Fe^2+^) can be converted into ferric (Fe^3+^) using Fenton reaction, and promotes ROS production and activates LOXs, simultaneously, which leads to oxidative damage (75, 76).

For CM, studies have indicated that there are elevated levels of ferritin in the CSF (23). Additional studies have suggested that ferritin is locally synthesized by brain cells, which reveals the involvement of ferritin is induced within the pathogenesis of CM in the brain. However, the specific mechanisms remain unclear (23). Furthermore, the increase of ferritin within the CSF is considered a measure to screen for meningitis (24). Compared to non-infectious neurologic disorders (i.e. seizure disorders and multiple sclerosis) and non-CM meningitis (i.e. viral and bacterial meningitis), the most significant increase of ferritin has been observed in CM (24). In this regard, compared to other types of meningitis, determining levels of CSF ferritin may be a diagnostic indicator, and CM may have a unique pathogenic mechanism involving iron metabolism. According to prior studies, the abundance of ferritin is a crucial factor that regulates sensitivity to ferroptosis (48, 50, 55). Ferritin is able to release iron into LIP through ferritinophagy, which causes increased sensitivity to ferroptosis. Therefore, this peculiar iron-dependent cell death is likely triggered by high levels of ferritin.

Iron accumulation is able to aggravate the progression of CM. A study has suggested that iron overload exacerbates experimental CM (77). Within the CM model constructed by two strains of *C. neoformans*, Sb26 (a-Dserotype) and Sb26Rev (a-D serotype), FeDx treatment distinctly increased brain colony forming units (CFU). Additionally, median survival times (MSTs) were markedly decreased (77).

Jarvis and colleagues analyzed the local and systemic immune responses in patients with HIV-associated CM and found that increased levels of proinflammatory cytokines and chemokines (i.e. IL-6, IFN-γ, and tumor necrosis factor (TNF)) are correlated with CSF macrophage activation, reduced fungal burden, rapid clearance of infection, and prognosis (78). However, these inflammatory cytokines are able to promote extracellular iron uptake by brain astrocytes and microglia by increasing the expression of the iron transporters divalent metal transporter 1 (DMT1) and Ferroportin 1 (FPN1) (79, 80). Moreover, current studies have validated that microglia have a vital function in controlling the growth of *C. neoformans* within the CNS (81). Consistent with prior work, IFN-γ plus lipopolysaccharide (LPS) enhanced anticryptococcal activity by the murine microglial cell line BV-2 (82). Nevertheless, through *in vitro* treatment using ferric nitrilotriacetate (FeNTA), the effect of iron-loaded brain microglial cells on stimulation of IFN-γ and LPS becomes significantly weakened (83). Therefore, excessive iron accumulation in the complex immune microenvironment of CM will affect effective cells’ function, but the specific pathway is still unknown.

Therefore, based on these results, we can preliminarily conclude that iron metabolism within the brain can be disturbed in CM, which mainly manifests by increasing ferritin levels within CSF and accumulating iron in the brain. These disorders may further cause physiological immune dysfunction of the microglia. However, whether iron-dependent ferroptotic cell death is involved in the above process needs further elaboration.

### Lipid Peroxidation Is a Common Manifestation of CM and Ferroptosis

Lipid peroxidation, a common hallmark of ferroptosis, is dependent on ROS (54, 56). ROS, which includes neutral molecules (i.e., hydrogen peroxide), ions (i.e., superoxide anion), or radicals (i.e., hydroxyl radicals), are mainly produced during cellular respiration and metabolic processes (84–86). ROS remain at homeostatic concentrations within mammalian cells and participates in specific normal physiological processes (86). In order to maintain the appropriate state of ROS, humans have various antioxidant defenses, which mainly include antioxidant enzymes (i.e. superoxide dismutase, catalase, and Gpx) and oxidation scavengers (i.e. vitamin E, ascorbic acid, carotenoids, and polyphenols) (86). However, ROS becomes excessively produced in certain diseases, and the antioxidants within the human body cannot keep them at their original homeostatic concentrations (54). ROS reacts readily with nucleic acids, proteins, and lipids at high concentrations, which eventually leads to DNA damage, protein denaturation, and lipid peroxidation (86). As oxidative stress is related to the processes of aging and cell death, ROS is involved in several age-related diseases (including AD, PD, and cardiovascular diseases), and these diseases are more or less confirmed to be related to ferroptosis (87–89). With regards to CM, there is also evidence of lipid peroxidation taking place with in brain cells.

Several studies have identified that *C. neoformans* is able to cause lipid peroxidation in the vasculature and the brain (25). In 2010, a study showed that cellular lipid peroxidation levels of rabbits inoculated with *C. neoformans* increased significantly, and histopathological examination indicated that the oxidative stress of multinuclear phagocytes mainly manifests as large amounts of granular brown pigment within cells (25). The study also found that *C. neoformans* increases lipid peroxidation in the rabbit model, largely due to macrophage-related oxidative stress and release of ROS (25). Not only that, but another animal experiment identified that thiobarbituric acid reactive substances (TBARS) in the brain are one of the most commonly utilized methods for measuring lipid peroxidation (26). The study discovered that, compared to the healthy group, lipid peroxidation levels in CM-infected rat brains were significantly increased (26). Analogously, when *C. neoformans* infects macrophages, significant levels of cellular lipid peroxidation occurs, and a higher volume density of dense lipid droplets within macrophages can be observed using an electron microscope (27). Studies that are related to HIV infection also provide evidence of lipid peroxidation and claim that lipid peroxidation may be linked to HIV-1-associated neurodegeneration (90, 91).

These results confirm the occurrence of lipid peroxidation in CM. However, overproduction of ROS and increased lipid peroxidation have severe impacts on cellular physiological functions, particularly within the CNS (92). Current studies have shown that high levels of ROS are able to react with PUFAs in various cellular membranes, which increases lipid peroxidation products, including 4-hydroxynonenal and malondialdehyde (93). These products have a long intracellular half-life and have been shown to have an effect on cellular activities that are related to neurite plasticity, which includes signal transduction pathways and ubiquitination (93). Additionally, an increasing number of neurological diseases have been shown to be related to increasing levels of lipid peroxidation and accumulation of lipid peroxidation products, such as neurodegenerative diseases (i.e., AD, PD, and amyotrophic lateral sclerosis) and CNS traumas (i.e. stroke, traumatic brain injury, and spinal cord injury) (92, 94, 95). Therefore, lipid peroxidation in CM can have an adverse effect on the CNS through the mechanisms mentioned prior.

In combination with the preceding descriptions of iron metabolism in CM, the mechanism of lipid peroxidation caused by *cryptococcal* infection of the brain is likely to, at least partly, include an iron-dependent process. However, this iron-dependent lipid peroxidation is a classic feature of ferroptosis. Hence, ferroptosis is likely involved in the potential link between lipid peroxidation and iron accumulation in CM. However, more systematic studies that analyze the function of ferroptosis in the pathogenesis of CM are needed in order to extend and validate these initial observations.

### CM Affects the Amino Acid Metabolism of the Brain

The effect of CM and *C. neoformans* on the host’s amino acid metabolism has not yet been fully elucidated. Only a few studies have discovered that CM has an effect on glutamate and cysteine metabolism, which is closely related to ferroptosis. Metabolomics research has identified several potential metabolic biomarkers of CSF that can help distinguish different types of meningitis. According to different studies, glutamate and cysteine may be potential metabolic markers of CSF that can help distinguish CM from tuberculous meningitis (96). Similarly, another study evaluated the metabolic status of lung epithelial cells that were infected with *C. neoformans*. Analysis of these results indicates that several pathways, including glutamate metabolism and GSH metabolism, were impaired at the low multiplicity of infections (MOI) samples, and that incubation at higher MOI resulted in the perturbance of the cysteine metabolism (97). Importantly, extensive research has proven that these dysregulated amino acids have a significant role in ferroptosis induction. Glutamate is an important sensitizer of ferroptosis (60). As described above, a high extracellular concentration of glutamate inhibits system $x_{c}^{-}$ and induces ferroptosis, which likely explains the toxic effects of glutamate when it accumulates to higher concentrations within the CNS (70, 71, 98). Indeed, animal experiments validated that glutamate, the neuroexcitatory amino acid, is involved in brain damage caused by *cryptococcal* infection (99). Researchers concluded that, as has been discovered with additional brain infectious diseases or toxic disorders, a depletion of glutamate in the brain of experimental murine cerebral cryptococcosis can be caused by excessive release of glutamate (99). However, accumulation of extracellular glutamate is thought to be a natural trigger for induction of ferroptosis (60).

Overall, although current research in amino acid metabolism of CM remains insufficient, combined with existing research and experimental results of iron metabolism and lipid metabolism, attention needs to be paid to the relationship between iron accumulation, lipid peroxidation, and dysregulated amino acid metabolism, all of which are hallmarks of ferroptosis. Hence, ferroptotic cell death is likely involved in the cellular damage of CM. Nevertheless, the current research remains inconclusive, and more in-depth research is needed.

## Immunomodulatory and Pro-Inflammatory Role of Ferroptosis

Since ferroptotic cells secrete DAMPs and alarmin, which are recognized by immune receptors and ultimately aggravate cell death and inflammation, ferroptosis is considered as both immunogenic and pro-inflammatory (100). In the model of folic acid-induced AKI, our study results concluded that ferroptosis can cause renal tubule damage by triggering inflammation, as upregulation of pro-inflammatory cytokines (interleukin-33, TNFa, MCP-1) and necroptotic proteins are quenched through Fer-1, a specific inhibitor of ferroptosis (101). Furthermore, inflammatory cells infiltrate into the ferroptotic tissue, which was noticeable, as determined by staining against F4/80, a 160 kD glycoprotein expressed by murine macrophages (61). Furthermore, ferroptosis regulates synchronized tubular cell death and contributes to immune-cell extravasation into the damaged tissue, while leukocyte transmigration and levels of pro-inflammatory cytokines are significantly decreased in the presence of Fer-1 (102). In an *in vivo* model of closed-chest myocardial ischemia-reperfusion injury (IRI), Fer-1 significantly reduced the initial infarct area and neutrophil infiltration into the heart, which suggests that immunogenicity and pro-inflammatory properties of ferroptosis are involved in early cardiomyocyte cell death and neutrophil recruitment *in vivo* (103). Results from this study also validated that ferroptotic cell death initiates neutrophil recruitment after heart transplantation through a TLR4/Trif-dependent pathway (103). These results reflect that ferroptosis can induce innate immunity and promote production of pro-inflammatory molecules in various diseases. However, the specific pathways through which ferroptosis carried out its immunogenic and pro-inflammatory effects have not yet been fully elucidated.

Arachidonic acid (AA) oxidation products, which are released from ferroptotic cells, are considered to be immunomodulatory signals and, therefore, regulate immunity. Eicosanoids are biologically active signaling lipids that are derived from AA and related PUFAs (104). Over the decades, inhibiting the formation or receptor-mediated actions of classical eicosanoids, such as through aspirin or other non-steroidal anti-inflammatory drugs, remains the prevailing strategy to alleviate inflammation. Friedmann Angeli et al. demonstrated that inducible Gpx4 disruption triggers Nec1-sensitive ferroptotic cell death, and determined that AA metabolites are released into the cell culture medium (61). Levels of eicosanoids, such as 5-hydroxyeicosatetraenoic acid (HETE), 11-HETE, and 15-HETE, were increased upon triggering of ferroptosis (61). Concurrently, ferroptosis-inducing agents (FINs) also induced a similar HETE signature (61). Analogously, in heart ferroptotic cells, the abundance of several HETE, epoxyeicosatrienoic acid (EET) species, and prostaglandin D2 were significantly increased, and Fer-1 treatment resulted in a decrease of these lipid mediators (103). Additional studies have also validated that inhibition of ferroptosis led to a reduction in production of pro-inflammatory lipid mediators, and inhibited TNF- or IL-1-mediated activation of the NF-κB pathway (105). These findings suggest that ferroptotic cells secrete specific lipid mediators that are involved in downstream signal transduction mechanisms.

Several studies have suggested that eicosanoids have an essential role in infection and inflammation and play a role in immunomodulation (106). For example, PGE2 leads to inhibition of necrotic cell death of macrophages, thus promoting pathogen resistance and host protection during *M. tuberculosis* infection (107). However, PGE2 impairs immunity to the influenza A virus by inhibiting macrophage antigen presentation and T-cell mediated immunity (108). Another example, despite the fact that definitive biological functions for HETE products have not yet been clarified, is that some may be ligands for peroxisome proliferator-activated receptor-α (PPARα) and PPARγ, which induce anti-inflammatory effects (109, 110). Therefore, pro-inflammatory lipid mediators secreted by ferroptotic cells can further regulate immunity or drive secondary inflammatory damage in ferroptosis-related pathologies.

In addition to lipid mediators, studies have also discovered that ferroptotic cells release high mobility group box 1 (HMGB1), which also mediates inflammation (111). HMGB1 is a nuclear protein that can be released by dead, dying, or injured cells (112). Once HMGB1 is released, it can bind to several receptors, including the toll-like receptor 4 (TLR4) and advanced glycosylation end-product specific receptor (AGER), in order to mediate immune responses (112). Thus, in order to measure the degree of HMGB1 release from ferroptotic cells, multiple cell lines, including HL-60 cells (a human leukemia cell line), HT1080 (a human fibrosarcoma cell line), PANC1 (a human pancreatic cancer cell line), and an immortalized MEF line, were treated with ferroptosis activators (erastin, sorafenib, RSL3, and FIN56) (111). Through ELISA analysis, it was discovered that HMGB1 released from these ferroptotic cells were significantly increased, and that this effect was hindered by ACSL4 knockdown and ferroptosis inhibitors, which include Fer-1, Lip-1, or baicalein (111). More importantly, this study demonstrated that ferroptotic cells are triggered by the pro-inflammatory cytokine TNF production in the bone marrow-derived macrophages, while anti-HMGB1 neutralizing antibodies are able to attenuate TNF production, which suggests that HMGB1 mediates inflammation response in ferroptosis (111). Therefore, inhibiting release of HMGB1 from ferroptotic cells is a potential anti-inflammatory strategy that is utilized for ferroptosis-associated diseases.

HMGB1 is a critical element that is required for immunogenicity of cancer cells. The absence of HMGB1 expression in dying tumor cells compromises DC-dependent T-cell priming by tumor-associated antigens (113). Moreover, Wang et al. confirmed a direct link between ferroptosis and anti-tumor immunity (114). As mentioned above, the system xc- is a disulfide-linked heterodimer that consists of SLC3A2 and SLC7A11 and plays a key role in ferroptosis. IFN-γ released from immunotherapy-activated CD8+ T cells downregulates SLC3A2 and SLC7A11 expression, and increases tumor cell lipid peroxidation and ferroptosis, which ultimately improves anti-tumor efficacy of immunotherapy (114). Therefore, regulating ferroptosis-associated metabolism in tumors is a potential strategy to improve the efficacy of cancer immunotherapy in the future.

For CM, confocal microscopy results indicated that *C. neoformans* induces translocation of HMGB1 in brain endothelial cells, which, in turn, results in endothelial cell damage and ultimately promotes breakdown of the blood-brain barrier (BBB) (115). Given the role of HMGB1 in inflammation and immune regulation, regulating ferroptosis may be an important method to suppress the outbreak of inflammation in particular infectious diseases, including CM.

## Ferroptosis and Infectious Diseases

As mentioned prior, ferroptosis is a type of programmed cell death that is characterized by free iron accumulation and toxic lipid peroxides. In infectious diseases, cell death is the most common phenomenon. Furthermore, it has been validated that ferroptotic cell death may be the main form of host cell death caused by specific pathogens. Additionally, ferroptosis of immune cells during infection is advantageous for infectious agents. Amaral et al. identified that *M. tuberculosis*-induced macrophage death *in vitro* is a type of necrosis, as opposed to apoptosis or pyroptosis, and that this form of cell death is accompanied by increased levels of intracellular labile iron and membrane lipid peroxides, as well as a decrease in GSH and Gpx4 expression levels (39). Interestingly, all of the significant parameters that were covered above are key hallmarks of ferroptotic cell death. Therefore, studies have investigated whether *M. tuberculosis*-induced death of macrophages is related to ferroptosis. In fact, results from these studies have confirmed that ferroptosis inhibitors, including pyridoxal isonicotinoyl hydrazone (PIH) and Fer-1, suppress lipid peroxidation and cell death, suggesting that ferroptosis may be involved in this form of necrosis (39). Importantly, these results were further confirmed by employing a mice model of *M. tuberculosis* infection (39). *P. aeruginosa*, a common gram-negative rod-shaped bacterium, is able to synthesize LOXs, which converts arachidonic acid in the membrane of the host cell to 15-hydroxyeicosatetraenoic acid, leading to the induction of lipid peroxidation and acting as an executor in ferroptosis (35, 36).

Similarly, another study discovered that macrophages infected with *Histoplasma capsulatum*, an environmentally-acquired fungal pathogen, can also lead to increased intracellular oxidation products, and eventually cell death (116). Despite the fact that the ferroptosis inhibitor Fer-1 can reduce the cell death caused by *H. capsulatum* infection, further experiments have validated that the mechanism decreases fungal ergosterol synthesis, instead of inhibiting ferroptosis (116). Consequently, upon exploring the relationship between ferroptotic cell death and pathogenic mechanism of pathogens, attention needs to be paid to ferroptosis inhibitors, especially Fer-1, which may have a direct inhibitory effect on pathogens. Furthermore, fungal infection can also be caused by plant ferroptotic cell death (117–119). Ceratocystis Fimbriata BMPZ13 infection of sweet potato leads to iron-associated ferroptotic cell death in leaves and veins (119). Incompatible rice (Oryza sativa)-Magnaporthe oryzae interactions induces ferroptotic cell death in rice cells (118).

At the end of 2019, coronavirus disease-2019 (COVID-19), caused by SARS CoV-2, started to spread worldwide. Several studies have further elaborated that cellular redox imbalance and iron incoordination can play an essential role in the pathogenesis of COVID-19 (41, 42). As an example, COVID-19 infection induces secretion of IL-6, which can stimulate ferritin and synthesis of hepcidin, eventually leading to iron dysregulation (40). Excess intracellular iron interacts with molecular oxygen in order to generate ROS through the Fenton reaction, which results in mitochondrial function disorder and ferroptotic cell death (40). A recent study also suggested that the mechanism of SARS CoV-2, leading to ferroptotic cell death, may be related to cysteine-associated cellular metabolism (42). Although the relative research appears sparse, these results suggest new potential links between ferroptosis and COVID-19, which may represent a novel strategy for treatment of COVID-19.

Moreover, with regards to HBV infection, liver fibrosis and hepatocellular carcinoma (HCC) are the most severe stages of disease progression (120). Hepatitis B virus X protein (HBx) plays a significant role in HBV replication and the development of HCC (43). Furthermore, HBx can further activate hepatic stellate cells (HSCs) by inhibiting ER stress, ferroptosis, and ultimately promoting liver fibrosis. In contrast, chrysophanol, which is derived from a Chinese herb, reverses this inhibition and may be a possible therapeutic strategy for treatment of anti-hepatic fibrosis (121). However, the relationship between HBx-induced ferroptosis and liver fibrosis needs to be further investigated.

In conclusion, these studies have indicated signs of ferroptosis across many infectious diseases; however, specific links need to be further researched. Existing evidence has validated that ferroptosis of immune cells during infection is beneficial to disease progression, and that ferroptotic cell death can induce release of DAMPs, which can both aggravate cell death and inflammation. Therefore, in order to control inflammation and cell death during infection, an effective regulation of ferroptosis may be a novel and possible strategy for treatment of infectious diseases.

## Discussion and Future Perspectives

Since the majority of infectious diseases involve well-characterized cellular necrosis and pro-inflammatory mechanisms, these represent possible new targets for therapeutic intervention. In this review, we elaborate on possible potential links between CM and ferroptosis. As iron overload and lipid peroxidation are fundamental characteristics of ferroptosis, these hallmarks are further reflected in CM. Hence, we reason that there might be a relationship between this cell death modality and CM.

**Figure 2:**
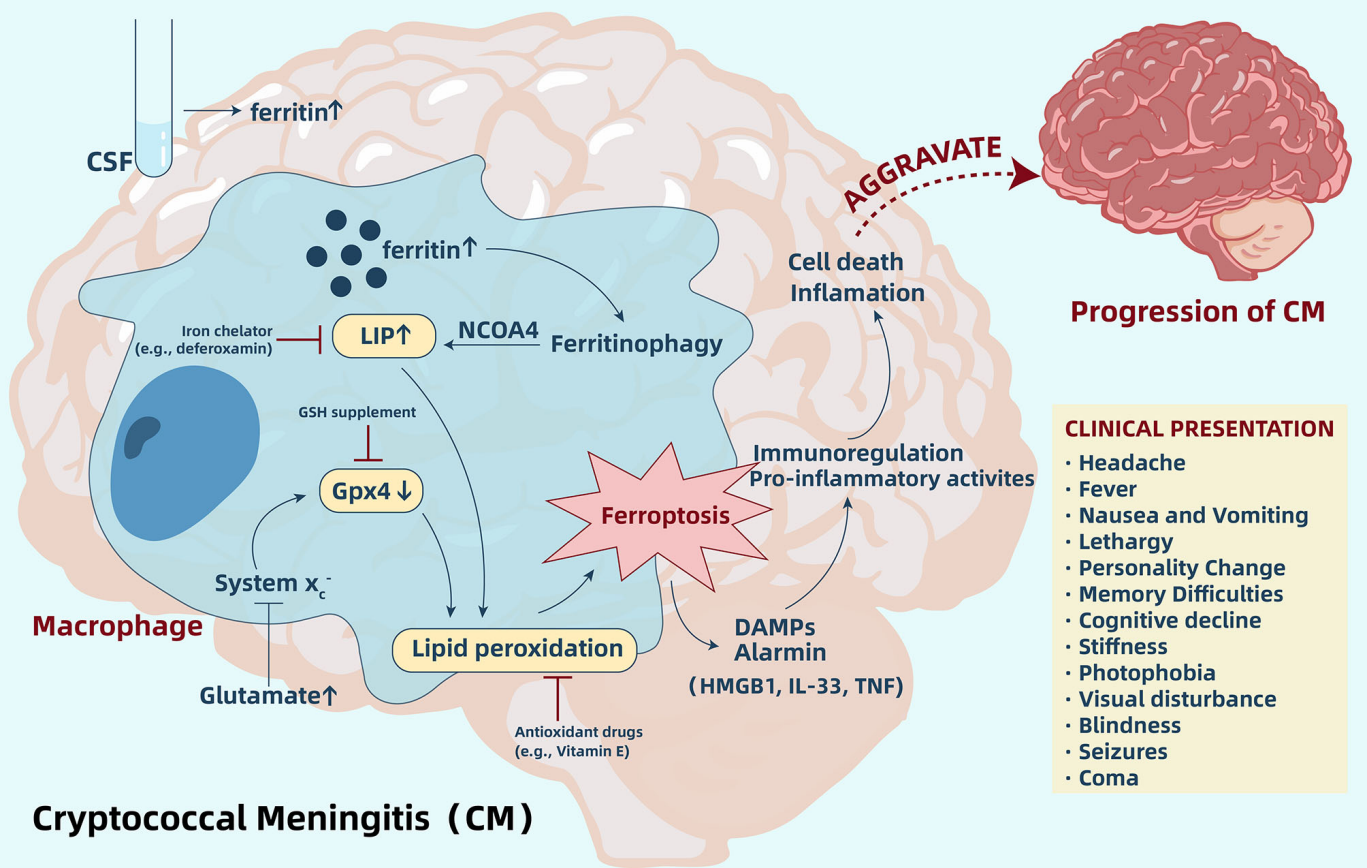
Ferroptosis aggravates cryptococcal meningitis (CM) by regulating immunity and pro-inflammatory activity. In CM, increased ferritin levels within the CSF and accumulation of iron in brain cells release iron into LIP through ferritinophagy, and increased glutamate may inhibit System xc-, which leads to a depletion of Gpx4 in cells. Accumulation of iron in LIP and depletion of Gpx4 induces lipid peroxidation and further triggers ferroptosis. Ferroptotic cells release DAMPs and alarmin, which participate in immune regulation and pro-inflammatory activities, ultimately aggravating cell death and inflammation. Regulating ferroptosis by inhibitors, such as iron chelators and antioxidants, may be a potential novel strategy to suppress the pathway and delay CM progression. CSF, cerebrospinal fluid; LIP, labile iron pool; GSH, glutathione; Gpx4, glutathione peroxidase 4; DAMPs, damage-associated molecular pattern molecules; HMGB1, high mobility group box 1; IL-33, interleukin-33; TNF, tumor necrosis factor.

Ferroptosis, a non-apoptotic form of cell death, is characterized by iron-dependent lipid peroxidation. For CM, an increase of ferritin within the CSF and accumulation of iron in the brain cells may indicate induction of ferroptosis. Ferritin’s abundance is a crucial factor that governs ferroptosis sensitivity as ferritin can release iron into the LIP through ferritinophagy, thereby causing increased sensitivity to ferroptosis (48–50). Importantly, intracellular iron accumulation and lipid peroxidation are able to coexist, and ferroptosis can exactly connect them together (**Figure 2**). Therefore, according to all current research results, we hypothesize that ferroptosis is likely involved in the pathogenic mechanism of CM, similar to other infectious diseases. However, we still need more in-depth research in order to validate this hypothesis. Through regulation of ferroptosis, we are able to better understand the relationship between iron accumulation and lipid peroxidation and ferroptosis. However, we need to pay more attention to the direct inhibitory effect of the regulator on pathogens. One study pointed out that Fer-1, a commonly used ferroptosis-inhibiting agent, reduced growth of *C. neoformans* in the YNB media by >95% (116). Hence, when designing an experiment, we should be able to regulate ferroptosis through multiple pathways.

In addition to ferroptosis, regulated necrosis is also related to pyroptosis and necroptosis. Recently, studies have identified that ferroptosis and pyroptosis may intersect (122). Therefore, in cellular necrosis in *C. neoformans* infection, it is necessary to exclude intersections with additional cell death pathways. Furthermore, ferroptosis is immunogenic and pro-inflammatory as ferroptotic cells release DAMPs and alarmin, which may be recognized by immune receptors and ultimately aggravate cell death and inflammation (100). Despite the fact that no studies have explored the expression of ferroptosis-associated DAMPs in CM, due to the extensive role of ferroptosis in the pro-inflammatory and immune regulation, this will also become a direction for future designed experiments. Overall, more systematic studies that analyze the function of ferroptosis in the pathogenesis of CM are needed in order to extend and validate these initial observations.

Moreover, iron accumulation may be an indicator of differential diagnosis of CM and helps estimate the severity of disease. Compared to non-infectious neurologic disorders and meningitis, other than CM, the increase in ferritin is most significant in CM, and iron overload can help exacerbate experimental CM and significantly weaken the function of microglia. Beyond that, overproduction of ROS and increased lipid peroxidation also severely impacts cellular physiological functions, particularly within the CNS cells. Therefore, the mechanism of iron metabolism and lipid metabolism in CM may be a potential new direction for future research.

Ferroptosis has been considered a novel target for therapeutic intervention. An increasing number of studies have established that ferroptotic cell death is closely related to numerous diseases, particularly in tumors. In recent years, the role of ferroptosis in infectious diseases has gradually emerged. As mentioned prior, *M. tuberculosis* induced iron accumulation and lipid peroxidation of macrophages and confirmed ferroptotic cell death, which can be suppressed by the ferroptosis regulator Fer-1 and iron chelation (39). *P. aeruginosa* utilizes host polyunsaturated phosphatidylethanolamines in order to induce theft-ferroptosis within the bronchial epithelium and may represent a possible therapeutic target against *P. aeruginosa*–related diseases (35). SARS CoV-2 may also trigger ferroptosis by promoting release of IL-6 and affecting cysteine metabolism, which suggests a novel strategy for COVID-19 treatment (40–42). This evidence indicates that ferroptosis is involved in pathogenesis of various pathogens, and inhibition of this unique form of cell death may be a novel treatment strategy. Unfortunately, there is no direct or indirect link between ferroptosis regulators and *C. neoformans* and CM. However, additional studies have been required to validate ferroptosis as a viable strategy for CM treatment. Nevertheless, prior to this, more research is needed to elucidate the status of ferroptosis in the pathological mechanisms of CM. In this review, we provide a promising research direction, and the potential to decrease tissue damage while reducing pathogenic burden is an attractive aspect of this research direction.

## Data Availability Statement

The original contributions presented in the study are included in the article/supplementary material. Further inquiries can be directed to the corresponding authors.

## Author Contributions

All the authors participated in analyzing and discussing the literature, commenting on, and approving the manuscript. AS and JS supervised the research, led the discussion, and wrote and revised the manuscript. All authors contributed to the article and approved the submitted version.

## Funding

This work was funded by the National Natural Science Foundation of China (81670567 and 81701144).

## Conflict of Interest

The authors declare that the research was conducted in the absence of any commercial or financial relationships that could be construed as a potential conflict of interest.
